# A Mismatch of Paradigms Disrupts the Introduction of Psycho-Educative Interventions for Families of Persons with SMI: An Interview Study with Staff from Community Services

**DOI:** 10.1007/s10597-018-0307-z

**Published:** 2018-08-14

**Authors:** Karin Persson, Margareta Östman, Karin Ingvarsdotter, Fredrik Hjärthag

**Affiliations:** 1 Department of Care Science, Faculty of Health and Society, Malmo University, 205 06 Malmö, Sweden; 2 Department of Psychology, Karlstad University, Karlstad, Sweden

**Keywords:** Severe mental illness, Family support, Psycho-pedagogic models, Municipality support

## Abstract

Treatment and support of people diagnosed with severe mental illness in Sweden takes place in out-patient psychiatric services or municipality services. Most of the responsibility for support in daily life are provided by the close family. One crucial matter is how to support these families. This research project aimed to investigate the Swedish construction with shared responsibility between county psychiatric care and municipality social care for consumers with severe mental illness affects actions in municipalities in relation to family support. Ten representatives from five municipality settings were interviewed. Five semi-structured interviews were analysed using a thematic analysis. The following themes emerged; One overarching theme, “a mismatch of paradigms”, and sub-themes: (a) “accentuating differences”, (b) “doubts about including the entire family in the same session” and (c) “lack of a uniform family support policy”. We conclude that a shared mandate needs a dialogue between psychiatric and municipality services concerning this mismatch.

## Introduction

In 2011 the Swedish National Board for Health and Welfare (NBHW) provided guidelines for treatment of schizophrenia. According to family interventions guidelines cover the aspects of treatment for which evidence exists, and were generated based on research from countries with entirely different structures of care. Evidence based on Swedish prerequisites is entirely lacking. To be able to accomplish a successful implementation of programs they need to be compatible with both organizational and professional norms (Greenhalgh et al. 2004) In this case the Swedish structure deviates from countries where evidence regarding psycho-educative programs is available. Two systems provide psychiatric care in Sweden, county council provides medical care and treatment, and the municipality provides support as economic support, accommodations and activities in daily life. Counties and municipalities have a mandate which obligates a shared responsibility for those consumers who need long-term support (Swedish National Board for Health and Welfare 2011). We therefore were interested in investigating how the Swedish construction with shared mandate affects the actions in municipalities in relation to family support.

Internationally and nationally guidelines are produced to secure and improve care. These guidelines mostly are underpinned of studies within a quantitative positivistic paradigm and advocates a more standardize care (Fisher and Happell 2009). In contrast to the constructivist paradigm, is predominant in social care. Were the work is based on individually established relationship with consumers, and through professional knowledge and experience develop skills for individuals to manage their illness. Within this paradigm the benefits of standardized care are questioned (ibid). Since ideas that fits with organization’s values and norms are more likely to be assimilated differences in funding values need to be considered in relation to the guidelines (Greenhalgh et al. 2004).

In Sweden, as well as in most of the western world, a majority of people diagnosed with severe mental illness (SMI), live in the municipality and receive treatment from out-patient psychiatric clinics or municipality services. Family members regularly assume a degree of responsibility for these persons’ care. These obligations have been described as burdensome and potentially disruptive to family members’ mental and physical health, social activities and financial situations (Awad and Voruganti 2008; Caqueo-Urízar et al. 2009; Flyckt et al. 2013; Hjärthag et al. 2010; Motlova 2007; Ostman and Hansson 2001, 2004; Ostman et al. 2005). Studies have demonstrated that families that includes consumers with SMI tend to be socially isolated (McFarlane et al. 2003; Ostman and Kjellin 2002). They show a scepticism to treatment programs in which social interactions are prominent, something that are at the core of the programs of psycho-pedagogical nature (Dixon 1999). While professionals are concerned with perceived conflicts between the philosophy and principles of psycho-pedagogic family models versus their agencies’ policies, consumers’ families have raised concerns about the model being too time-consuming, too focused on medical interventions and too concerned with preventing relapses (Dixon 1999). They preferred a model centred on family well-being (Dixon et al. 2000).

Multifamily interventions, which address consumers and their relatives from more than one specific family, may improve these families’ situations by creating a more positive family environment and lowering the consumer’s risk of relapses, thus reducing the burden on relatives (Dyck et al. 2002; Henken et al. 2007; Justo et al. 2007; Lucksted et al. 2012; Pharoah et al. 2010). Several studies have shown that the effectiveness of multifamily interventions depended not on the model used but on the length of the intervention (a period of at least 9 months was found to be most effective) (Abramowitz and Coursey 1989; González-Blanch et al. 2010; Hansson et al. 1992; Orhagen and D’elia 1992; Smith and Birchwood 1987). Despite the proven effectiveness of longer-term interventions, difficulties when implementing these findings have led to a search for ways to incorporate methods developed into daily praxis in psychiatric treatment and in municipality services (Barbui et al. 2014; Wollersheim et al. 2005). However, the gap between what is known about effective treatment and what is being offered to consumers and their families’ remains very wide (Proctor et al. 2009; Tansella and Thornicroft 2009).

In specialised out-patient care services run by municipalities, there is a need for personnel to be able to provide care and support for consumers with SMI and their families. Also, co-operation and support from psychiatric services is regarded as crucial to the success of municipality programs. In the last years there has been a development of peer-to-peer family programs run by municipalities (Lucksted et al. 2013; Rexhaj et al. 2017). These programs have shown improved satisfaction for relatives of consumers with SMI in their daily lives, independent of professional support (Lucksted et al. 2013; Pickett-Schenk et al. 2008; Rexhaj et al. 2017).

In Sweden, clinical experience with multifamily interventions in municipalities is lacking. Therefore, a national randomised multicentre family intervention study was planned for both psychiatric services and municipality services (Ingvarsdotter et al. 2015). The multifamily intervention was based on a model by McFarlane (2004); it evaluated the effects of a 12-month intervention consisting of 22 sessions involving both consumers and their families. The main part of the sessions consisted of providing the informants with both guidelines for managing the illness and tangible training in solving problems created by the illness (ibid.). That intervention study will be designated in this paper as “the proposed study” and the model will be designated “the proposed model”.

The present study investigated how the Swedish model with shared responsibility for consumers with SMI between county psychiatric care and municipality social care affects actions in municipalities in relation to family support.

## Methods

We used a qualitative design in which semi-structured interviews with personnel from multiple municipalities were conducted, transcribed and then evaluated using a thematic analysis (Braun and Clarke 2006).

### Sampling Frame

This study is part of a research project of which the aim was to evaluate the potential of introducing a psycho-educational multifamily model in Sweden for persons suffering from SMI and their families (Ingvarsdotter et al. 2015). Of the various municipal centres from the middle and south of Sweden that were asked to take part in the research project, five of six declined. In accordance with the UK Medical Research Council guidelines for complex interventions (Craig et al. 2008), we decided to conduct a series of interviews as part of a feasibility study aimed at developing a more effective future intervention. Representatives from five municipal centres were invited to be interviewed about family interventions in general and especially about the proposed model, which they had chosen not to try, all five centres agreed to participate. The participating centres had from 12,000 to 100,000 inhabitants in their municipalities. The individual informants were six men, and four women. Their ages ranged between 45 and 65, and each of them had been organising family support or supporting consumers with SMI in their municipalities for a minimum of 5 years. They were social service staff and nurses’ aides; and nurses. At least one of the informants had prior experience working in a psychiatric department.

These municipalities were chosen because representatives had earlier been in contact with the research group during meetings or conferences were research concerning family burden and the need for family support had been presented. When the recruitment started contact was established with these representatives, who later became the informants. The informants had in their former contact with the research team expressed an interest of improving family support in their municipalities. All, except two, had the authority to accord or refuse participation in the project. The two who didn’t have the authority to decide about participation had a mandate to prepare the case and participate in discussions that preceded the decision about participation in the project or not.

Following reasons were given for declining: two centres were interested but after they had invented their population they found it too small to be able to regularly recruit groups over time. One centre declined with the reason that the intervention could not be seen as a municipality obligation. Another two centres were interested but declined for the reason that, if the intervention should be successful it needed to be done in cooperation with the psychiatric service in their county, who when questioned by the research group declined to participate.

### Data Collection

In order to better incorporate clients’ perspectives into the study design, an already-available trained user advisory board participated in the research and design process. This board consisted of consumers of mental health services and the relatives of such users. Throughout the study, the board gave useful comments on both the layout process and the questionnaire as well as advice on effectively interacting with the study’s informants.

We designed a semi-structured interview guide with open-ended questions. The questions aimed to elicit informants’ opinions on how the proposed model of psycho-educational multifamily group treatment compared to their own organisations’ programs; the questions also sought their views on the potential of implementing the proposed model. In addition, informants were asked about their thoughts and processes before and after their municipality decided not to take part in the proposed study. The interview guide gave the researcher the opportunity to ask follow-up questions. Our opening question was: tell me what got you interested in the model? Probing questions like “what did you think of that?” was asked to further deepen the answers.

Five interviews were conducted between December 2013 and April 2014 with a total of 10 informants taking part. One of the interviews involved a single participant, another included three informants and the remaining three involved two informants each. .The authors, who had psychiatric multi-professional experience, conducted all of the interviews, which took place at the informants’ workplaces during business hours. All interviews were conducted in Swedish and audio-recorded with the consent of each participant. All authors performed and transcribed at least one interview each. The interviewers transcribed their interviews for analysis, and the data collected comprised 53 pages of written transcript.

### Analysis

Transcripts were reviewed using a thematic analysis patterned after the five step process described by Braun and Clarke (2006). First all of the authors read all of the transcripts and discussed their content briefly. Next, the first author reread the material and developed initial ideas about items of potential interest. Then, the initial coding process was done by the first author. Next step was to developed potential themes, and check themes through another re-reading of the transcripts. At the fourth step all authors discussed the presented potential themes in relation to the transcripts and the support of data for each theme. The content of the themes were organized until they were found valid and discussion continued until consensus was reached. Finally themes were named as follows “Accentuating differences” and “Doubts about including the entire family in the same session” and finally **“**Lack of a uniform family support policy”. An overarching theme, “A mismatch of paradigms”, was found to represent all data. Finally, quotations from each participant were linked to the speaker by number to assure anonymity.

## Results

The main theme which emerged from our interviews was that “a mismatch of paradigms” places obstacles in the way of introducing psycho-educative interventions for families with consumers with SMI. There were three subthemes: (a) “accentuating differences”, (b) “doubts about including the entire family” and (c) “lack of a uniform family support policy”.

### A Mismatch of Paradigms

Our informants experienced difficulties with obtaining, between the municipality services and psychiatric services, a clear and explicit understanding of which service was responsible for providing family support. In their experience, the two services’ policies were founded upon different paradigms. For municipality services, the primary goal, in line with the constructivist paradigm, was to support an individual’s autonomy. However, in psychiatric services, in which our informants perceived that a positivistic paradigm prevailed, the focus seemed to be on evaluating treatment interventions. The tension between these two approaches was seen as an obstacle to introducing family interventions into municipality services.

### Accentuating Differences

All informants agreed that providing support to consumers with SMI involved addressing numerous aspects of daily life and that successful support needed to include efforts from both psychiatric and municipality services. Informants believed that, since the family intervention model was psycho-educational, its treatment activities were hard to merge with their services’ philosophy. This conflict led informants to accentuate the differences between municipality and psychiatric services’ practices. They all felt uncertain about whether or not it was appropriate to support a multifamily model, and all had reservations about the proposed family support model. For instance, training in solving problems was viewed by some informants as a treatment activity not suitable to municipality services. They also thought it inappropriate for a municipal service to inquire which relatives the municipality service should invite to join group sessions, and were unsure of what criteria should be used to do so. The informants declared that such activities should be the duty of psychiatric services, through which everyone is aware of a consumer’s actual diagnosis. Furthermore, they stated that the large number of group sessions in the proposed model could make the group members more familiar with each other, and then it was more likely that subjects outside of municipality’s range of responsibilities might arise; examples included medication and other aspects of treatment.

*We cannot engage in a dialogue about medication that does not work because then we can only say that you have to bring that question up with your outpatient psychiatric service. We work with the healthy parts*. [Informant 2]

Some informants claimed that, according to the Social Services Act, municipalities’ social services should not be involved in any activity referred to as treatment. Instead, support from social services should embrace the total life situation of the individuals involved; this is in contrast to psychiatric services, which (according to informants) today only was concerned with the treatment of the consumer. Support offered by municipality services, according to most informants, tended to pay attention to the individual as a human being, whereas in the case of psychiatric services, consumers were viewed as diagnoses rather than as human beings.

*The outpatient psychiatry is unwilling to include the family. They only care about the patient, even if the patient is not capable of taking care of himself*. [Informant 6]

Some of our informants felt that, when the National Guidelines on Psychosocial Interventions were released for implementation in 2011, municipality services were not included in the transition process from the beginning. If the process have been done otherwise, the different views of family support between the municipality and psychiatric services could have been highlighted in order to facilitate future cooperation. They claimed that, since the municipalities and the psychiatric services were not involved on an equal footing from the start, the ability of municipalities to establish cooperation in this area was hindered.

Even if some informants said they were used to cooperate and sharing responsibility with psychiatric services, when it came to family support, they felt that these services had abandoned them. The psychiatric services provided treatment and support without any coordination with municipality services.

The informants wondered if the multifamily group model presented to them, and such models in general, were suitable for their organisations. If the sessions included both families and consumers at the same time, our informants thought they should be undertaken by psychiatric services. They also expressed doubts about models founded upon a diagnostic perspective. For them, social work should address a person’s need for support in a variety of areas, based on an assessment of the person’s abilities and disabilities and not solely on a psychiatric diagnosis. Such a perspective was described by our informants as rehabilitation-oriented and marked a difference in their approach to that of psychiatric treatment. They emphasised the importance of attempts to increase the individual’s participation and self-determination, considering a client’s individual wishes to be an essential part of their recovery. Social work, from their point of view, should provide individual support and training in skills that enable individuals to cope with daily life on their own.

*We already work with relatives in our model* […] *We don’t want our work to include group sessions*. [Informants 2 and 3]

### Doubts About Including the Entire Family in the Same Session

This theme reflects disagreement over whether entire families should be included in interventions. Although some informants had reservations about the feasibility of a multifamily model run by municipal services, all stated that providing support to relatives of consumers was an issue that municipal services should address. However, several informants mentioned factors that made them uncertain about the way in which support to family members ought to be given. One concern was that the target group, consumers with SMI, was too small to warrant the expenditure of resources that the model required. Another was the potential difficulty of locating relatives of adults with SMI. Also, most informants considered it advisable to allow relatives to talk about the difficulties they were experiencing when the consumer in question was not present.

*They have to talk about their burdens, and that is where family support begins*. [Informant 1]

Most of the informants wished to focus on their clients as individuals and were against considering the entire family to be part of their responsibility. Some informants related experiences of consumers who needed to distance themselves from their parents; reasons for this included, for example, their desire to achieve the autonomy of living an independent life without the close involvement of their families. For them, the proposed model including group meetings with the entire family had the potential to cause discord between family members, especially between parents and adult children.

*We are working to strengthen individuals’ autonomy and ability to make their own decisions, perhaps without parental involvement*. [Informant 1]

Opinions about when it would be appropriate to invite relatives to participate in a multifamily model differed among informants, even those from the same organisation. Several felt that such an offer should be given soon after the onset of the illness. They considered such early efforts helpful in getting over a consumer’s initial crisis and that the model ought to be run by psychiatric services. Some informants expressed scepticism towards family interventions offered more than 2 years after a person was diagnosed. One participant imagined what a relative might say if support was offered so late:

*When my relative became ill, that is when I needed the support* […] *It needs to be given early in the course of the disease!* [Informant 4]

### Lack of a Uniform Family Support Policy

This theme included informants’ attitudes as to which aspects of family support they considered indispensable. There was no consensus about including these elements in a model or about whether they required a model at all. Opinions diverged on this topic between informants from different municipalities and even those from different departments in same organisation. The informants experienced that a constant reorganisation of the services paved the way for short-term policies related to the organisation of family support within the municipalities. Both the lack of a uniform policy on family support and the introduction of new policies were mentioned as problematic in the municipalities, although these situations allowed space for individual solutions. As a result, family support in a given municipality seemed more related to individual preferences and staff members’ experience than to organisational policy.

*We also have our own rehabilitation model; I use it a lot in these groups. The last group, which just started, we call “recovery and rehabilitation”*. [Informant 8]

Most informants viewed municipality services as addressing of all the needs and capacities of their clients as individuals. A majority of the informants also felt that a considerable part of the interventions currently offered by municipalities was increasingly specialised and fragmented and lacked a holistic perspective. They thought this placed the additional burden of coordinating various activities on consumers and their families. They stressed the need for these families to have someone who could help them keep track of all their activities and contacts. A coordinator, for instance a case manager either from the psychiatric service or the municipality service, was needed who would take charge of the families’ interest and act as a bridge between the different services—someone who could look at a family’s overall situation and help coordinate the various interventions.

*Those families who don’t have a case manager definitely need one. When someone in a family has an SMI, everything is disrupted*. [Informant 11]

In some of the interviews, discussions took place between informants about the possibilities that family interventions offer. Some claimed that a user model earlier proved successful would more easily detect general patterns and needs linked to consumers that includes the entire group. Continuing to regard consumers with SMI as individuals—a perspective consistent with the philosophy of municipal agencies—could be a hindering factor in this systematisation. Other informants had different ideas about the kind of family support services that municipalities should offer. They felt that help was important even at the later stages of an illness. They also believed that a family support model could be the result of cooperation between the municipality and psychiatric services. Some informants thought it was important to offer help and give hope when the lives of consumers with SMI and their families had changed. Those informants favoured some kind of support that would allow family members to meet people in other families who are in the same situation. Learning from each other and having fun together, these informants thought, would be helpful and could provide hope for the future.

*Relatives need information about the disease, but also the hope that it might actually improve. Another aspect* [of SMI treatment] *is that an ill person might recover, and just hearing that has to be fantastic*. [Informant 11]

Some informants who already followed a certain plan feared that a fixed model would have a negative impact on the method they already used. However, they seemed amenable to adopting parts of other methods or models. Several informants mentioned shared decision-making as an element that had recently been assimilated into existing methods.

While some of the informants wanted to retain the types of support that are currently offered, others expressed the wish for innovation and changes in the services they currently provided. They advocated a more open attitude towards different ways of meeting the needs of the target group. Informants from multiple municipalities suggested “open” services in which relatives could attend some sessions without committing themselves to attend others. They also proposed that voluntary organisations might collaborate and run joint groups together. For example, they were guardedly open to inviting psychiatric services to arrange guest lectures about various diagnoses.

*Cooperating with voluntary organisations would probably be possible, but doing so with a psychiatric service from the county would be difficult*. [Informant 5]

## Discussion

The professionals from municipality services interviewed in this study believed that a mismatch of paradigms in psychiatric and municipality services creates obstacles to introducing psycho-educative interventions for families that include consumers with SMI.

We encountered difficulties in recruiting participating municipality centres when we tried to test an evidence-based multifamily intervention model that had earlier proven to be effective. This finding indicates a gap between the existing culture of delivering support to consumers with SMI and what is expected of the same organisations by national guidelines, such gaps have earlier shown to be hindering factors for family support interventions (Cohen et al. 2010; Dixon et al. 2001, 2014; Gold et al. 2006). Although research has shown that family interventions work in psychiatric research settings, only 2–8% of consumers with SMI and their families are offered organised family interventions in psychiatric settings (Rummel-Kluge et al. 2006). The number of families offered such interventions by municipality social services is, to the best of our knowledge, unknown.

The model offered in our study did not coincide with the ideas of municipality services about what should be included in family support. This issue was central when the informants made the decision to partake or not. A similar situation has been reported by Dixon, who encountered resistance against introducing psycho-educative family support in any care or support agency other than in-patient psychiatric services (Dixon et al. 2001).

Our informants experienced differences in the fundamental values of psychiatric and municipality services. Foremost for municipality services was the holistic perspective including the preservation of each person’s right to be treated as an individual. This was considered to be at odds with involving other family members. Despite their loyalty to their organisations, most of our informants said that they would be willing to participate in the proposed family support intervention if ways to effectively cooperate with the psychiatric services in their regions could be found. Many believed that family support attracted little interest among the administrators of psychiatric services, although they stated they had never vented this fact with the psychiatric services. Earlier studies have concluded that leaving this issue to professionals’ discretion, rather than requiring family support, has been a barrier to implementing such support in psychiatric services (Cohen et al. 2010; Dixon et al. 2001).

While most of our informants had had some experience of collaborating with psychiatric services with regard to consumers, none had spoken with psychiatric services staff about differences in the founding values of their respective organisations, e.g. they had never discussed how their different policies in questions about involving families could be united. In times such as these, when resources in all organisations providing social services and medical care are strained, new ways of providing family support are needed to overcome such hindering factors.

Internationally stakeholders in community mental health services seems to experience difficulty trying to create more effective ways of supporting relatives of consumers with SMI. Their efforts have been promoted by the recovery movement, which has stressed the need to provide support to consumers and their families (Browne et al. 2008; Farkas et al. 2005; Fisher and Happell 2009; Gordon and Ellis 2013). Some of our informants had ideas about new ways of offering support, which they proposed should be less strictly arranged. These new ideas were in harmony with the tendency to shift the responsibility for family support onto voluntary organisations and onto models led by family members themselves; such models have been tested with good results (Lucksted et al. 2013; Pickett-Schenk et al. 2008).

However, according to our findings, it is doubtful if there is a willingness to accept a new family support model not in line with present values in their practice. They adopt to the bottom up perspective and “let it happen”, especially since the recommended guidelines did not reflect their practice (Greenhalgh et al. 2004; Sandström et al. 2014). Thus, organisations are neither ready to free up time nor to offer incentives to provide family support Barriers to the implementation of family support models continue to exist, although the models’ advantages have been clearly demonstrated (Cohen et al. 2010). Leaving these decisions to professionals rather than making them mandatory has deprived relatives of consumers of the support that could improve their lives and the lives of their ill family members.

### Methodological Considerations

This study is, to our knowledge, one of the first conducted in a municipality setting—certainly the first in a Swedish context—investigating hindering factors among municipality professionals to implementing support models in their departments. One strength of this study is the cooperation between the researchers and the advisory board throughout the process. A further strength is that the analysis phase was conducted in a collaborative manner, with the multi-professional team of authors working together to reach a consensus.

One limitation of this study is that, though it involved only informants from centres that declined to take part in the intervention study, its sample might have been larger under optimal research circumstances. Another limitation is that the study did not fulfil the implementation process of the proposed model for complex interventions as recommended by the Medical Research Council (Craig et al. 2008). This should be done in future studies. The choice to mix interviews done with one informant and group interviews may have affected the results. It was decided with respect for the services who already had put an considerable amount of time in this project in order to save time for the organizations. Even though there were some interactions between some of the informants, the question-responses mode were emphasized not the interaction. Qualitative studies mostly use a small sample size, this study might have given a too narrow picture, and thus the results might not be not transferrable to other contexts. It is also possible that bias have been induced in the analysis, since all informants were truly engaged in family support for consumers. We have tried to counter-balance this with a solid analyse of our results in relation to the validity of the data.

## Conclusion

Professionals in municipality agencies express concern regarding family interventions in families including consumers with SMI. The obstacles to accepting such interventions seem rooted in professionals’ experience of the differing fundamental values of psychiatric and municipality services. Another obstacle lies in the organisational structure, which has resulted in a shared mandate and different obligations between these services. In the face of this stalemate, there is an urgent need to establish a dialogue between psychiatric and municipality services concerning the mismatch of existing paradigms in order to facilitate an introduction of family interventions in municipality services.
